# DFT Insights into Noble Gold-Based Compound Li_5_AuP_2_: Effect of Pressure on Physical Properties

**DOI:** 10.1021/acsomega.3c01217

**Published:** 2023-04-20

**Authors:** Gokhan Surucu, Aysenur Gencer, Ozge Surucu, Md. Ashraf Ali

**Affiliations:** † Department of Energy Systems Engineering, Gazi University, Ankara 06500, Turkey; ‡ Department of Physics, Karamanoglu Mehmetbey University, Karaman 70100, Turkey; § Department of Electrical and Electronics Engineering, Atilim University, Ankara 06836, Turkey; ∥ Department of Physics, Chittagong University of Engineering and Technology (CUET), Chattogram 4349, Bangladesh

## Abstract

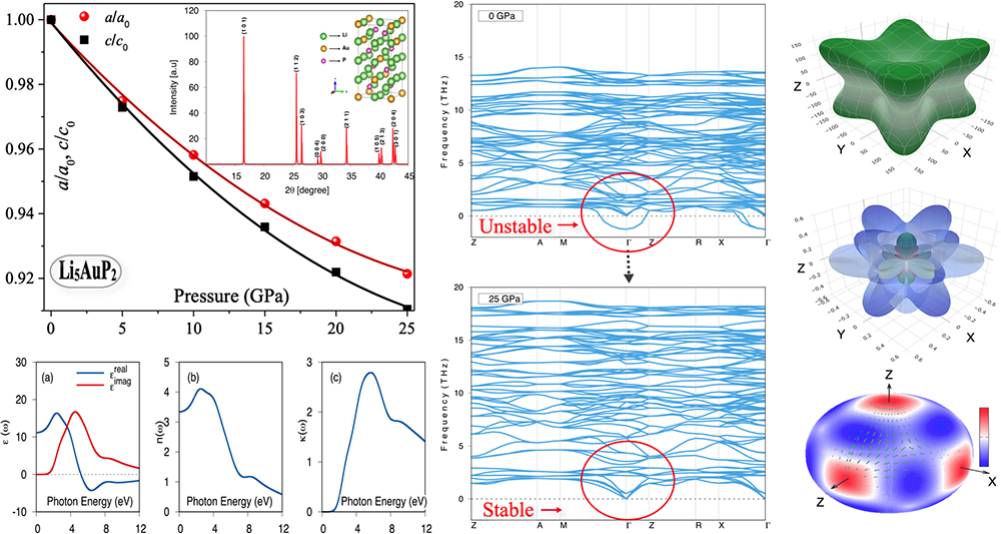

In this study, the Li_5_AuP_2_ compound is investigated in detail due to the unique chemical properties of gold that are different from other metals. Pressure is applied to the compound from 0 to 25 GPa to reveal its structural, mechanical, electronic, and dynamical properties using density functional theory (DFT). Within this pressure range, the compound is optimized with a tetragonal crystal structure, making it mechanically and dynamically stable above 18 GPa and resulting in an increment of bulk, shear, and Young’s moduli of Li_5_AuP_2_. Pressure application, furthermore, changes the brittle or ductile nature of the compound. The anisotropic elastic and sound wave velocities are visualized in three dimensions. The thermal properties of the Li_5_AuP_2_ compound are obtained, including enthalpy, free energy, entropy × *T*, heat capacity, and Debye temperature. The electronic properties of the Li_5_AuP_2_ compound are studied using the Perdew–Burke–Ernzerhof (PBE) and Heyd–Scuseria–Ernzerhof (HSE) functionals. The pressure increment is found to result in higher band gap values. The Mulliken and bond overlap populations are also determined to reveal the chemical nature of this compound. The optical properties, such as dielectric functions, refractive index, and energy loss function of the Li_5_AuP_2_ compound, are established in detail. To our knowledge, this is the first attempt to study this compound in such detail, thus, making the results obtained here beneficial for future studies related to the chemistry of gold.

## 1 Introduction

Intermetallic compounds consisting of two or more metal/metalloid elements have unique structures, various stoichiometries, and mixed bond types (e.g., ionic, covalent, metal, and van der Waals bonds), which give them exceptional physical and chemical properties. For this reason, these compounds are considered applicable for a wide range of technological purposes, for example, catalysis, hydrogen storage, electron transport, nonlinear optics, shape memory, and decoration.^1,2^ The hardness, luminescence, and superconductivity attributes of these compounds also pay the way for other applications in the industry.^1,2^

Among the intermetallic compounds, the gold-based ones have an exceptional place due to the fascinating properties of the gold (Au) itself; in particular, the chemical properties of gold differ from other metals in some respects. The primary variations arise from relativistic effects, which play a significant role in chemical bonding and the oxidation state. The high oxidation states, such as +3 and + 5, are favorably affected by the expansion of the Au 5d orbital, causing the Au 6s orbital to contract. This special condition allows gold to have very high electronegativity and oxidation states; in alkali-metal aurides, this value is −1.^3−7^ Additionally, under pressure, the oxidation state of gold can be expanded to higher levels—+4, +6, and even beyond 3—serving as a 6p-block component.^3,8^ Another point is that Au features different intermolecular interactions and chemical bonding with other transition metals. Due to the aurophilic attraction between the Au^+^ centers caused by the relativistic effect and electronic correlation of close-shell components, Au compounds offer intriguing features and a wide range of uses.

On the other hand, during compression, the 6s lone pair of Au^–^ can be activated to engage in bonding, which causes a remarkable structural change in the CsAu molecule, changing it from the CsCl type to the Cmcm phase. In addition, P, S, and I are three nonmetal elements with similar electronegativity that can form covalent bonds with gold as a transition metal. Au^3+^ is a planar and fourfold configuration, whereas Au^+^ frequently displays a linear pattern with twofold configuration. Respectively, these formations are known as sp hybridization and dsp^2^ hybridization. Moreover, having a covalent tetrahedral configuration in Au, such as an sp^3^ hybridization, is very rare due to the absence of a 6p electron. In the literature, it is shown that Au can acquire a 6p-element characteristic by gaining electrons from the covalent tetrahedral configuration of other atoms with an sp^3^ hybridization.^3,8^ It has also been reported that the reaction of Li with Au and P under high pressure leads to extraordinary negative oxidation of Au. The interaction of Li, P, and Au at high pressure stabilizes the compounds with unique bonding patterns and features owing to the wider stoichiometry and synergetic charge transfer of ternary compounds.^7^ In that same work, Zhang et al.^7^ revealed exciting properties, such as sp^3^ hybridizations and bonding patterns, for the Li_5_AuP_2_ compound at 25 GPa.

In the present work, the authors aimed to investigate the physical attributes related to the Li_5_AuP_2_ compound in detail—namely, structural, mechanical, dynamical, and opto-electronic properties—under pressure between 0 and 25 GPa. To the best of our knowledge, so far there has not been an attempt on such a large scale to investigate the Li_5_AuP_2_ compound. Apart from finding that the compound is stable at 25 GPa, Zhang et al.^7^ did not mention any other information about its stability at lower pressures. To do so, in this paper, the phonon dispersion curves are calculated at lower pressures (0 to 20 GPa), leading to the detection of imaginary frequencies in the phonon dispersion up to a pressure of 18 GPa. At 19 GPa, the phonon dispersion curve exhibits all the positive frequencies, indicating its stability at this pressure level. However, to reveal the consequence of the applied hydrostatic pressure, the physical properties calculated at 0 to 25 GPa (with an interval of 5 GPa) are presented. Given these contributions to the literature, it is hoped that this study can serve as a referencing point for other attempts in the future by experts in this field, as well as a guideline to use this compound for technological applications in various sectors.

## 2 Computational Details

In this study, the Li_5_AuP_2_ compound is investigated using the Cambridge Serial Total Energy Package (CASTEP)^9,10^ based on density functional theory (DFT). The exchange correlations are considered within the generalized gradient approximation (GGA) using the Perdew–Burke–Ernzerhof (PBE) functional.^11^ The structural optimizations are performed using the Broyden–Fletcher–Goldfarb–Shanno (BFGS) technique,^12^ and the density mixing is taken as Pulay mixing^13^ for the electronic structure self-consistent calculations. The cutoff energy is employed as 800 eV, and the **k**-points are sampled using the Monkhorst–Pack grid^14^ with 6 × 6 × 3 **k**-points. The valence electron configurations for the Li, Au, and P atoms are taken as 1s^2^2s^1^, 5d^10^6s^1^, and 3s^2^3p^3^, respectively. The Li_5_AuP_2_ compound is optimized using total energy, maximum force, maximum displacement, and maximum stress tolerances as 5 × 10^–6^ eV/atom, 0.01 eV/Å, 5 × 10^–4^ Å, and 0.02 GPa, respectively. The electronic structures are also studied with hybrid functionals using the Heyd–Scuseria–Ernzerhof (HSE06) method^15^ to identify the electronic band gaps, which are closest to the experimental values. The dynamical properties are considered with the phonon dispersion curves, which are obtained using density functional perturbation theory (DFPT).^16^ The supercell is chosen as 2 × 2 × 2 with 800 eV cutoff energy and 2 × 2 × 1 **k**-points for the calculation of the phonon dispersion curves. Furthermore, the thermodynamic properties are determined with the quasi-harmonic Debye model^17^ using the phonon dispersions. The anisotropic elastic properties are obtained using the ELATE software.^18^ The sound wave velocities of this compound are obtained using the Christoffel tool^19^ that solves the Christoffel equation.^20^ The effect of pressure on the unit cell structure of Li_5_AuP_2_ is revealed by plotting the normalized values of the lattice constants (*a*/*a*_0_ and *c*/*c*_0_), and the change of *a*/*a*_0_ and *c*/*c*_0_ are fitted to demonstrate the pressure effect on the axial direction by the following equations:
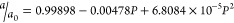
1
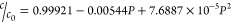
2

The coefficient of *P* is the compressibility along the *a*-axis (or the *ab* basal plane) and the *c*-axis (or the interlayer), respectively. These coefficients are obtained from Figure 2, which will be explained in the next section.

## 3 Results and Discussion

### 3.1 Structural Properties and Stability Considerations

**Figure 1:**
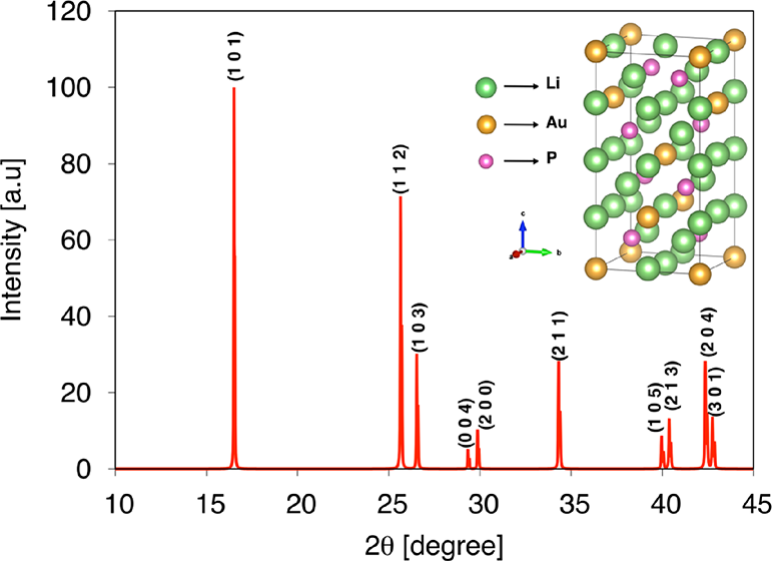
The crystal structure and X-ray diffraction pattern of the Li_5_AuP_2_ compound.

Figure 1 shows the tetragonal crystal structure of the Li_5_AuP_2_ compound with the space group I-42d (S. G. No.-122). The Li atoms are at the 4b, 8c, and 8d Wyckoff positions, while the Au atoms are at the 4a position and the P atoms at the 8d Wyckoff position. The crystal structure of the Li_5_AuP_2_ compound is optimized at zero temperature and zero pressure. Figure 1 shows the X-ray diffraction (XRD) pattern for this compound at 0 GPa. The maximum peak is at 16.51° for the (1 0 1) direction. After this ground-state optimization, pressure is applied to the compound up to 25 GPa with an interval of 5 GPa. The obtained lattice parameters, volume, and final enthalpy values are listed in Table 1 for the different pressure values applied. As can be concluded from Table 1, the lattice parameters decrease with increasing pressure as expected due to the application of hydrostatic pressure.

**Table 1:** Optimized Lattice Parameters (*a* and *c* in Å), Volume (*V* in Å^3^), and Final Enthalpy (*E*_f_ in eV)

pressure	*a*	*c*	*V*	*E*_f_
0 GPa	5.977	12.168	434.811	–1718.723
5 GPa	5.831	11.844	402.710	–1717.421
10 GPa	5.728	11.582	379.962	–1716.203
15 GPa	5.640	11.389	362.282	–1715.046
20 GPa	5.570	11.217	347.985	–1713.940
25 GPa	5.506	11.076	335.770	–1712.874

**Figure 2:**
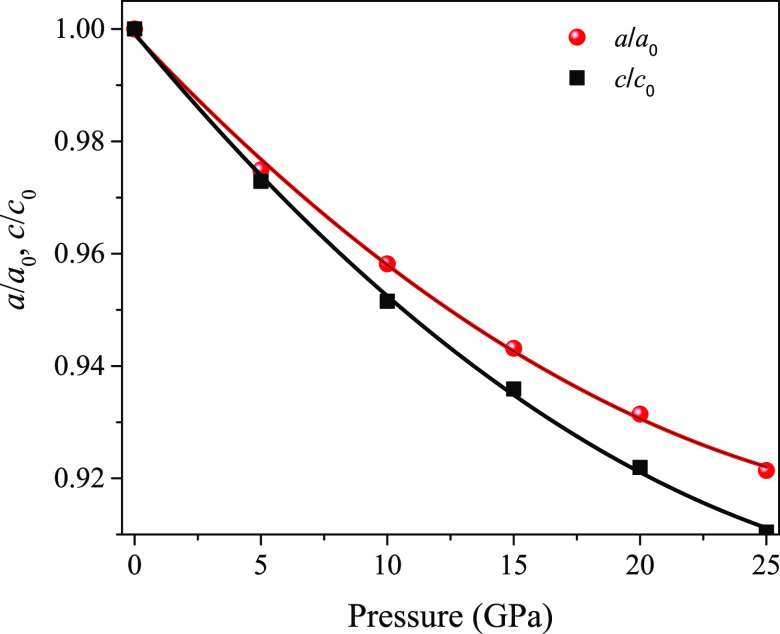
Variation of normalized lattice parameters with pressure.

The effect of pressure on the unit cell structure of Li_5_AuP_2_ is investigated by plotting the normalized values of the lattice constants (*a*/*a*_0_ and *c*/*c*_0_), as shown in Figure 2. The lattice constant, *a* (*c*), is seen to decrease to a 7.85 (8.95) % smaller value of 5.51 (11.08) Å at 25 GPa from 5.98 (12.17) Å at 0 GPa. As shown in Figure 2, the slope of *c*/*c*_0_ is greater than that of *a*/*a*_0_, indicating greater compressibility along the *c*-axis than the one along the *a*-axis. Moreover, it is seen that d(ln *a*)/d*P* = 0.00478 GPa^–1^ is 1.13 times smaller than d(ln *c*)/d*P* = 0.00544 GPa^–1^, indicating more sensitivity of *c* than *a* to pressure.

After the structural optimizations, the elastic constants are determined to reveal the mechanical stability of the Li_5_AuP_2_ compound at different pressures. This compound has six independent elastic constants—namely, *C*_11_, *C*_12_, *C*_13_, *C*_33_, *C*_44_, and *C*_66_—required for tetragonal crystal structures. Table 2 lists the elastic constants calculated using the stress–strain method to satisfy the Born stability criteria,^21^ as given in eq 3, for 0 GPa. The elastic constants are also seen to increase with the pressure increment. The pressure-dependent Born stability criteria is given in eq 4. All the calculated elastic constants for all pressures satisfy the Born stability criteria, as concluded in Table 2. This clearly shows that the Li_5_AuP_2_ compound is mechanically stable up to 25 GPa pressure.
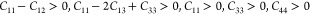
3
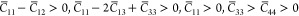
4where *C̅_ii_* = *C*_11_ – *P* (*i* = 1,3), *C̅*_1*i*_ = *C*_1*i*_ + *P* (*i* = 2,3).

**Table 2:** Calculated Elastic Constants (*C_ij_* in GPa) for the Li_5_AuP_2_ Compound at Different Pressures

pressure	*C***_11_**	*C***_12_**	*C***_13_**	*C***_33_**	*C*_44_	*C*_66_
0 GPa	77.24	44.53	47.04	74.07	61.58	60.39
5 GPa	104.70	62.13	64.54	97.19	77.62	75.17
10 GPa	130.32	79.56	81.54	120.90	90.19	87.84
15 GPa	153.48	96.63	97.36	145.29	100.45	97.38
20 GPa	176.14	113.10	113.04	167.10	109.31	106.01
25 GPa	197.57	129.18	128.08	188.29	118.00	113.61

**Figure 3:**
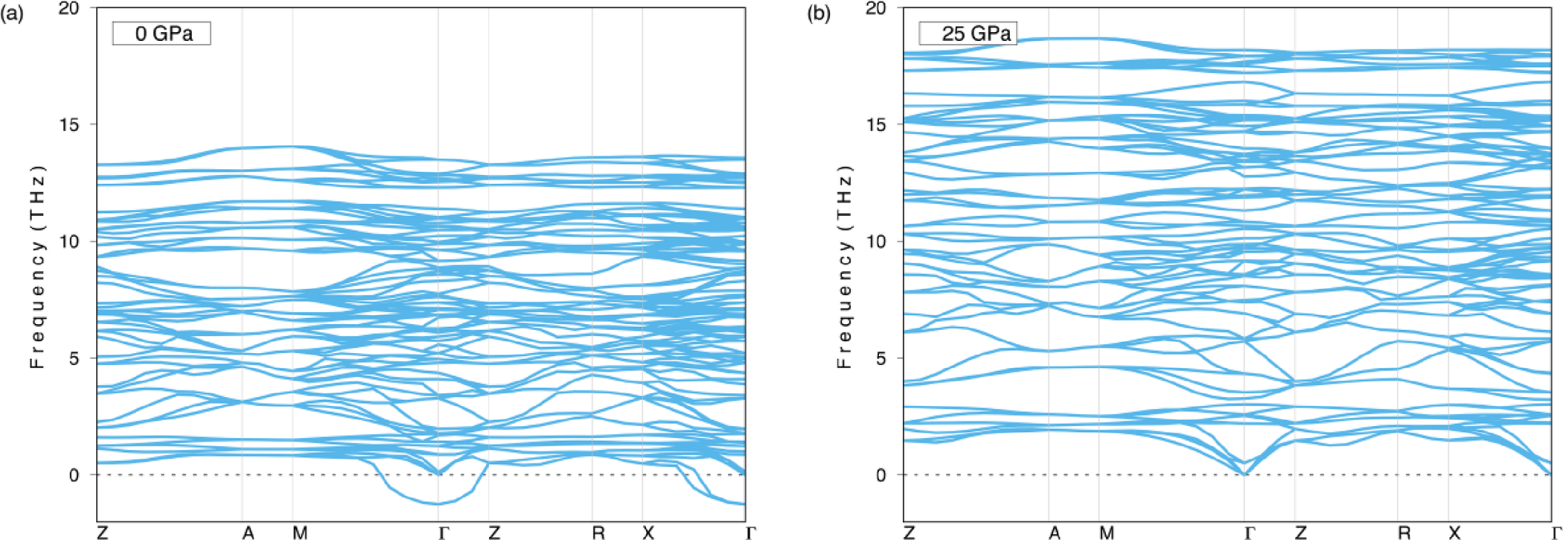
Phonon dispersion curves of the Li_5_AuP_2_ compound at (a) 0 GPa and (b) 25 GPa.

**Figure 4:**
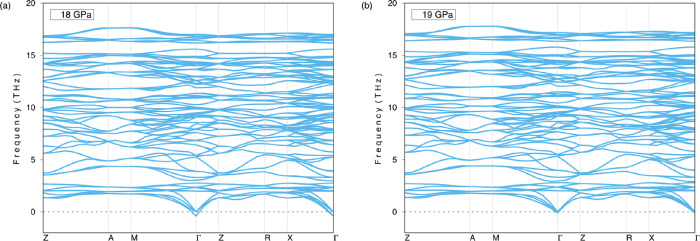
Phonon dispersion curves of the Li_5_AuP_2_ compound at (a) 18 GPa and (b) 19 GPa.

Dynamical stability is another issue to be considered with respect to compounds. In this respect, the Li_5_AuP_2_ compound is assessed by determining the phonon dispersions using DFPT.^16^Figure 3 shows the phonon dispersion curves for pressures at 0 and 25 GPa. As shown in Figure 3a, the Li_5_AuP_2_ compound is dynamically unstable due to the negative frequencies in the phonon dispersion curves at 0 GPa; however, Figure 3b shows that the compound is dynamically stable with no negative frequencies in the phonon dispersion curves at 25 GPa pressure, where the phonon frequencies are seen to increase. The phonon dispersion curves for 5, 10, 15, and 20 GPa are given in Figure S1 in the Supplementary file, where the compound is shown to be dynamically stable only at 20 GPa. After these calculations, the pressure is lowered from 20 to 15 GPa to find out whether the compound is dynamically stable at any other values between these two. Figure 4 shows the phonon dispersion curves at 18 and 19 GPa where the compound can be seen as unstable at 18 GPa and stable at 19 GPa. As a result, it can be concluded that Li_5_AuP_2_ is dynamically stable at pressures between 19 and 25 GPa.

### 3.2 Mechanical Properties

The mechanical properties of the Li_5_AuP_2_ compound are considered in detail since they are essential for the technological applications of any material. The estimated elastic constants shown in Table 2 are used to derive the bulk modulus, shear modulus, and other polycrystalline parameters. These properties are determined using the Voigt,^22^ Reuss,^23^ and Hill^24^ approximations. The Voigt approximation gives the upper values, while the Reuss approximation gives the lower values for a polycrystalline property. The Hill approximation is the average of the Voigt and Reuss approximations, and the results are closer to the experimental ones. The resistance to shape change when hydrostatic pressure is applied is known as “bulk modulus”. The Li_5_AuP_2_ compound has a bulk modulus of 56.20 GPa at 0 GPa. Table 3 lists the bulk modulus values at higher pressures. As the pressure increases, so does the bulk modulus of the compound. “Shear modulus” is defined as the resistance to shape change at a constant volume. The shear modulus of the compound is 34.97 GPa at 0 GPa and lower than its bulk modulus, which means that it is more resistant to shape change under hydrostatic pressure. Apart from this, shear modulus increases with the pressure increment, as listed in Table 3. Young’s modulus is the resistance to elastic deformations under tension or compression. Among these moduli, Young’s modulus is the highest, with 86.88 GPa at 0 GPa; as such, the Li_5_AuP_2_ compound is more resistant to elastic deformations. In addition, the pressure increment results in a higher Young’s modulus for the Li_5_AuP_2_ compound. The Poisson’s ratio (ν), defined as the amount of perpendicular expansion or contraction when compression or stretching is applied, is a crucial factor in determining a material’s bonding nature. Generally speaking, materials have dominantly ionic bonding when ν is around 0.25 and covalent bonding when ν is 0.10.^25^ The Li_5_AuP_2_ compound has dominantly ionic bonding with a Poisson’s ratio of 0.24, and this ratio increases with the pressure increment, as listed in Table 3. The *G*/*B* ratio can also be applied to determine the bonding type of a material, and the 0.6 value for this ratio indicates a dominantly ionic bonding. In comparison, a 1.1 value for the G/B ratio indicates a dominantly covalent bonding. The Li_5_AuP_2_ compound has a dominantly ionic bonding with a *G*/*B* ratio of 0.62 at 0 GPa, which is consistent with the results of the Poisson’s ratio. Additionally, it is seen that the *G*/*B* ratio decreases when the pressure increases, as listed in Table 3.

**Table 3:** Calculated Bulk Modulus (*B*, in GPa), Shear Modulus (*G*, in GPa), Young’s Modulus (*E* in GPa), Poisson’s Ratio (ν), *G*/*B* and *B*/*G* Ratios, and Cauchy Pressure (*P*_a_^Cauchy^ and *P*_b_^Cauchy^) for the Li_5_AuP_2_ Compound at 5, 10, 15, 20, and 25 GPa Values

pressure	*B*	*G*	*E*	ν	*G*/*B*	*B*/*G*	*P*_a_^Cauchy^	*P*_b_^Cauchy^
5 GPa	76.53	44.21	111.21	0.26	0.58	1.73	–13.08	–13.04
10 GPa	96.26	52.15	132.51	0.27	0.54	1.85	–8.65	–8.28
15 GPa	114.95	58.99	151.12	0.28	0.51	1.95	–3.09	–0.75
20 GPa	133.03	64.92	167.52	0.29	0.49	2.05	3.73	7.09
25 GPa	150.40	70.49	182.89	0.30	0.47	2.13	10.08	15.57

The *B*/*G* ratio is obtained to determine the ductility or brittleness of the Li_5_AuP_2_ compound, which is found brittle with a 1.61 value at 0 GPa, lower than 1.75. The Li_5_AuP_2_ compound remains brittle at 5 GPa with a *B*/*G* ratio lower than 1.75, and it is ductile at 10, 15, 20, and 25 GPa with a *B*/*G* ratio higher than 1.75, as listed in Table 3. The Cauchy pressure can also be used to determine the ductility or brittleness of a material. For tetragonal structures, the Cauchy pressure is defined as *P*_a_^Cauchy^ = *C*_13_–*C*_44_ and *P*_b_^Cauchy^ = *C*_12_–*C*_66_ and a positive (negative) value for the Cauchy pressure indicates the ductility (brittleness).^26^Table 2 lists the calculated Cauchy pressure values for this compound under different pressures, showing that it is brittle under 5, 10, and 15 GPa while it is ductile under 20 and 25 GPa. As can be seen, the results of the *B*/*G* ratio and the Cauchy pressure are conflicting for 10 and 15 GPa values. The *B*/*G* ratio generally yields consistent results for cubic crystal systems because the bulk modulus is the resistance to shape change under hydrostatic pressure. However, the Cauchy pressure depends on *C*_13_, *C*_44_, *C*_12_, and *C*_66_, providing information based on the behavior of the material in different orientations. As listed in Table 3, the *B*/*G* ratio and the Cauchy pressure provide consistent results for 20 and 25 GPa, at which pressure levels the compound is stable both mechanically and dynamically.

**Figure 5:**
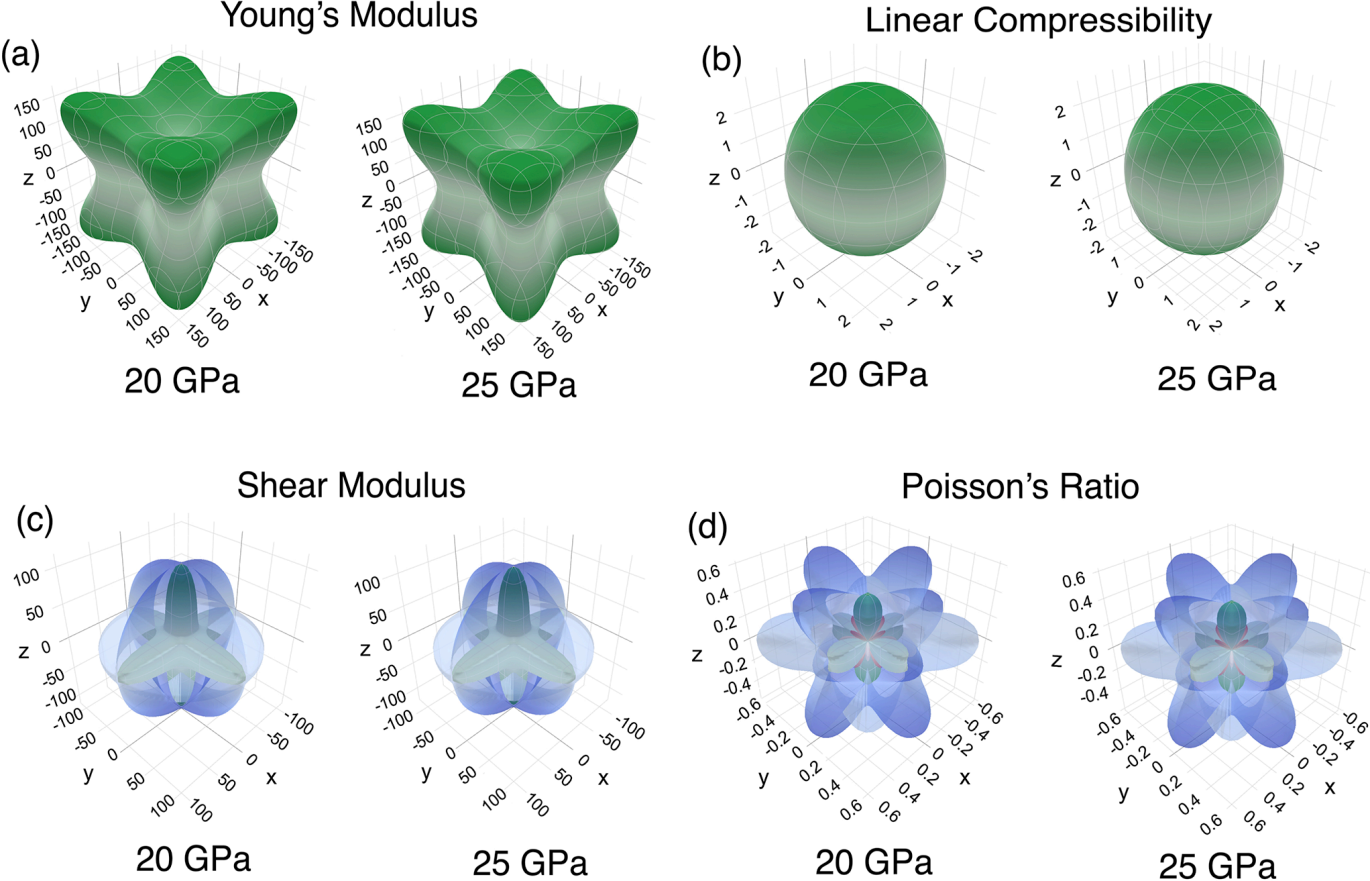
Direction-dependent polycrystalline properties— (a) Young’s modulus, (b) linear compressibility, (c) shear modulus, and (d) Poisson’s ratio—of the Li_5_AuP_2_ at 20 and 25 GPa values.

The anisotropic elastic properties are considered for materials related to the development of microcracks, dislocations, etc.^27^ Understanding these properties is essential to enhance the mechanical durability of materials.^28^Figure 5 shows the anisotropic elastic properties of the Li_5_AuP_2_ compound under 20 and 25 GPa values, where it is found to be dynamically stable. Furthermore, the anisotropic elastic properties of this compound under 0, 5, 15, and 20 GPa values are given in Figure S2 in the Supplementary file. In this figure, spherical shapes indicate isotropy, whereas distorted shapes indicate anisotropy for that polycrystalline property. According to Figure 5 and Figure S2, the Li_5_AuP_2_ compound is anisotropic for Young’s modulus, linear compressibility, shear modulus, and Poisson’s ratio. In terms of linear compressibility, the compound is isotropic under 0 GPa. The green and blue shapes correspond to the minimum and maximum values of that polycrystalline property, respectively, while the red shapes represent negative values. There are negative values only for the Poisson’s ratio for this compound. Similar to auxetic materials, a negative value for the Poisson’s ratio indicates perpendicular expansion when stretching is applied. Table 4 lists the minimum and maximum values for the polycrystalline parameters mentioned earlier under different pressure values. According to this table, the minimum and maximum values for Young’s and shear moduli increase with the pressure increment, whereas the values for the linear compressibility and Poisson’s ratio decrease with the pressure increment.

**Table 4:** Minimum and Maximum Values of Young’s Modulus (*E* in GPa), Linear Compressibility (β), Shear Modulus (*G*), and Poisson’s Ratio (ν)

	Young’s modulus		linear compressibility		shear modulus		Poisson’s ratio	
pressure	*E*_min_	*E*_max_	β_min_	β_max_	*G*_min_	*G*_max_	ν_min_	ν_max_
0 GPa	37.71	134.67	5.88	6.03	14.21	61.58	–0.32	0.84
5 GPa	47.25	176.63	4.14	4.79	18.04	77.62	–0.30	0.86
10 GPa	57.53	204.80	3.26	3.88	21.86	90.19	–0.27	0.86
15 GPa	69.49	231.49	2.76	3.19	25.93	100.45	–0.24	0.83
20 GPa	78.74	255.34	2.37	2.77	29.22	109.31	–0.22	0.82
25 GPa	87.88	277.77	2.10	2.46	32.37	118.00	–0.22	0.81

**Figure 6:**
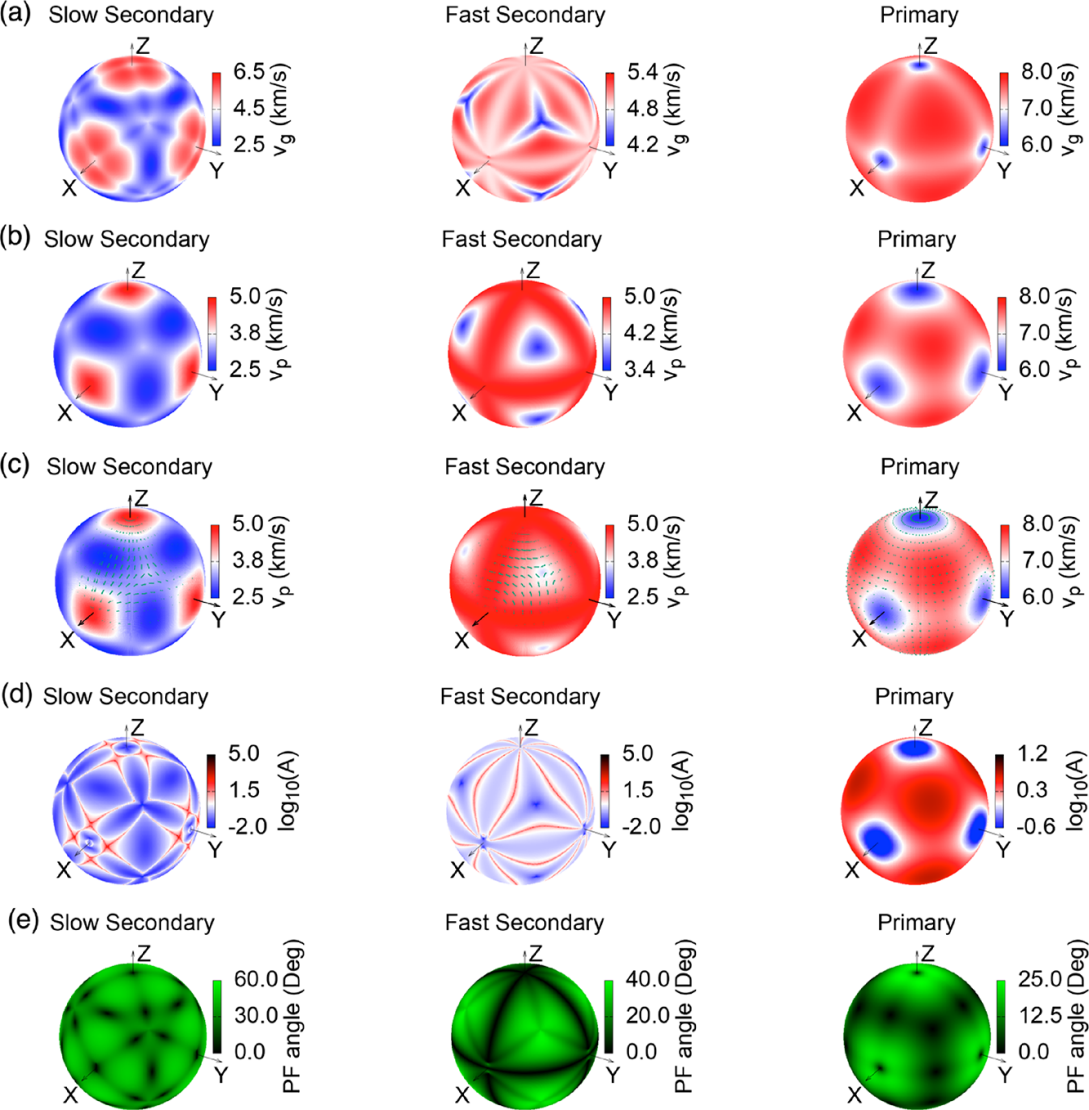
(a) Group velocity, (b) phase velocity, (c) polarization of the sound waves, (d) enhancement factor, and (e) power flow angle of the Li_5_AuP_2_ compound.

The sound wave velocities of the Li_5_AuP_2_ compound were obtained using the Christoffel tool and by determining the elastic stiffness matrix. Figure 6 shows the group velocity, phase velocity, phase polarization, enhancement factor, and power flow angle of Li_5_AuP_2_ at 20 GPa. The respective figures for the above features at 25 GPa are given in Figure S3 in the Supporting Information. Two transverse velocities are given as fast secondary and slow secondary phases for the sound wave velocities. In contrast, the figure exhibits a longitudinal velocity as the primary phase. Group wave velocity, shown in Figure 6a, has higher values along the *x*, *y*, and *z* directions for the fast and slow secondary phases, while the primary phase has lower values along these directions. Phase velocity has a similar behavior to group wave velocity, as shown in Figure 6b. Phase polarization, shown in Figure 6c, is transverse polarization in the primary phase, followed by longitudinal polarization in the secondary phases. The enhancement factor, defined as the ratio of the direction of the group wave velocity to the direction of the phase wave velocity, has lower values along the *x*, *y*, and *z* directions in both the primary and secondary phases, as shown in Figure 6d. The power flow angle is the angle between the group wave velocity and the phase velocity. Figure 6e shows the power flow angle with low values along the *x*, *y*, and *z* directions. Figure S3 shows similar behaviors to those shown in Figure 6 under the 25 GPa pressure value.

**Figure 7:**
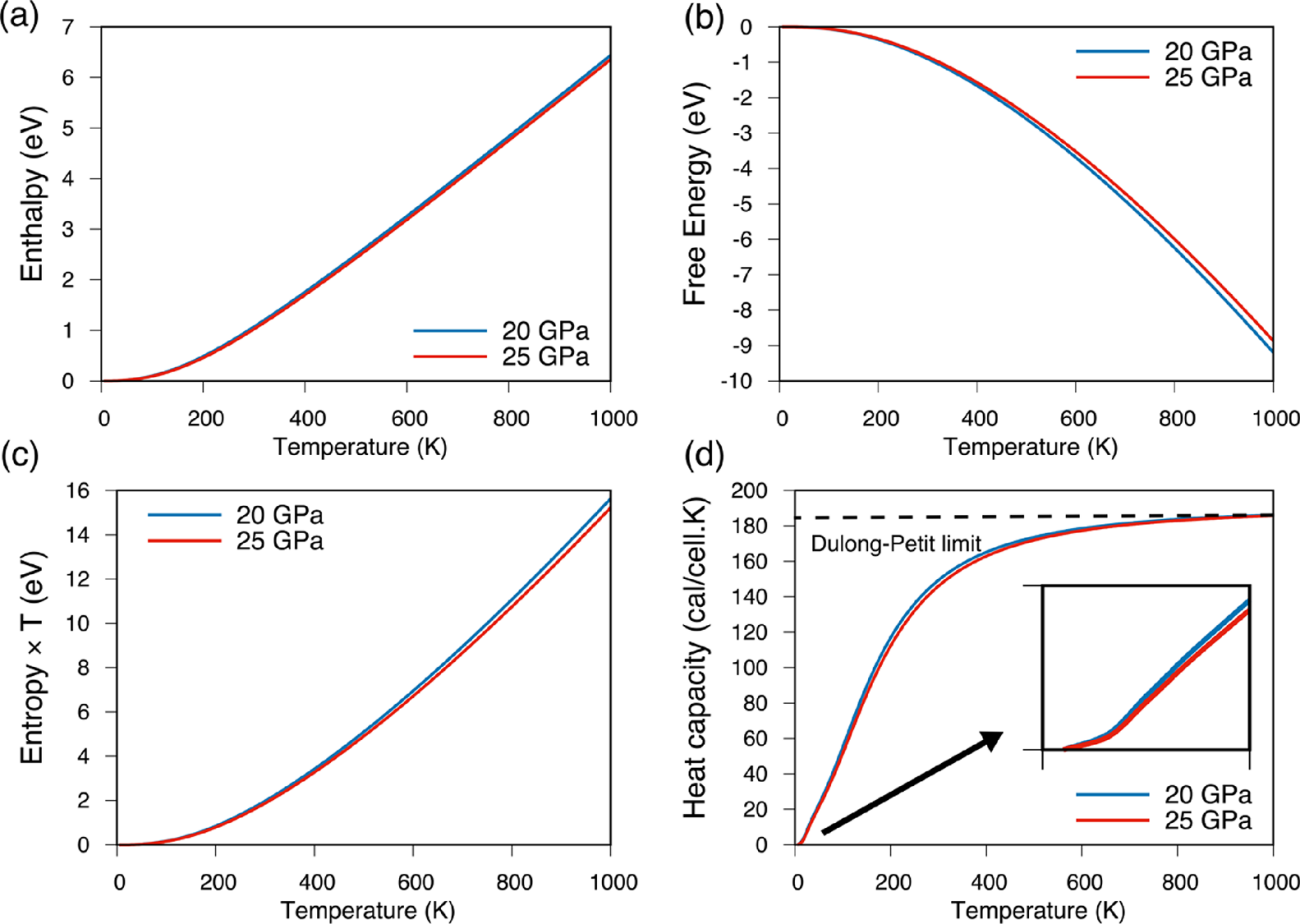
The temperature dependence of (a) enthalpy, (b) free energy, (c) entropy × *T*, and (d) heat capacity of the Li_5_AuP_2_ compound at 20 and 25 GPa values.

The thermal properties of the Li_5_AuP_2_ compound were calculated in the range of 0 to 1000 K using the quasi-harmonic Debye model. Figure 7 shows the temperature dependence of enthalpy, free energy, entropy × *T*, and heat capacity of the Li_5_AuP_2_ compound at 20 and 25 GPa values. As can be concluded from Figure 7, the enthalpy and entropy × *T* increase while the free energy decreases as the temperature increases. For the heat capacity, there is a sharp increase at low-temperature values. Additionally, at high-temperature values, the heat capacity reaches a constant value as 3*nR*, where *n* is the number of atoms in the crystal structure of the compound and *R* is the universal gas constant. This constant value for the heat capacity is known as the “Dulong–Petit limit”. In addition, the inset shows the heat capacity behavior between the 0 and 50 K temperature range. Furthermore, the enthalpy, free energy, and entropy × *T* have higher values at 25 GPa than at 20 GPa, while the heat capacity is higher at 20 GPa than at 25 GPa.

**Figure 8:**
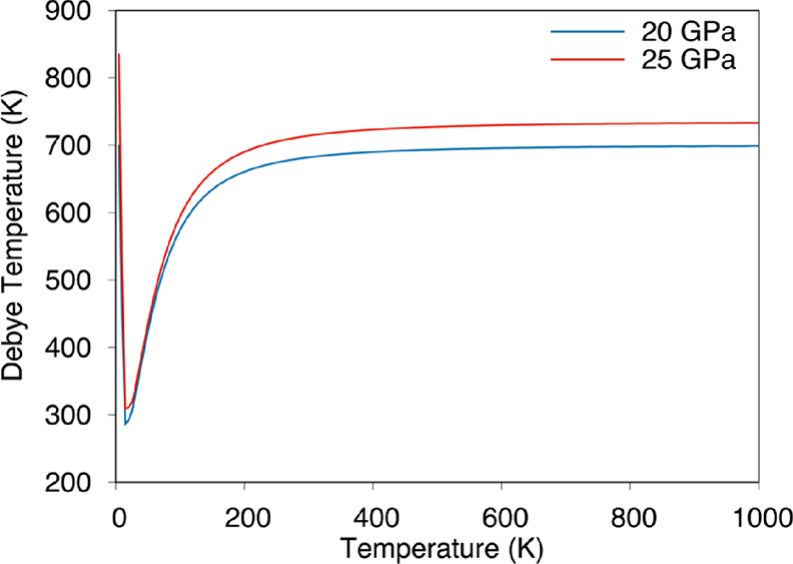
Temperature dependence of the Debye temperature for the Li_5_AuP_2_ compound at 20 and 25 GPa.

The Debye temperature is related to the physical properties of a material, such as specific heat and melting temperature. If a material has a high Debye temperature, it indicates high thermal conductivity.^30^Figure 8 shows the temperature dependence of the Debye temperature of the Li_5_AuP_2_ compound at 20 and 25 GPa values. The Debye temperature values are 698 and 732 K at 20 and 25 GPa, respectively, at high temperatures, as shown in Figure 8. In addition, there are dips around 15 K with the Debye temperature values as 286 and 308 K at 20 and 25 GPa, respectively.

### 3.3 Electronic and Optical Properties

**Figure 9:**
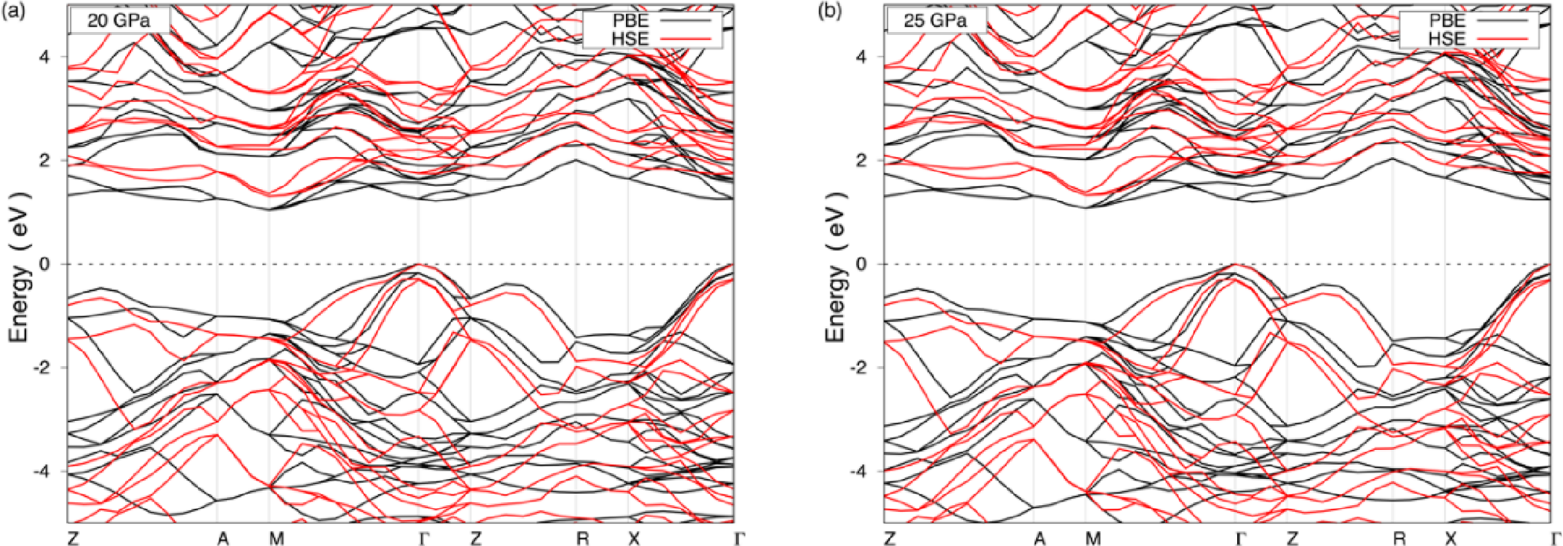
Electronic band structure of the Li_5_AuP_2_ compound at (a) 20 GPa and (b) 25 GPa using the PBE and HSE methods.

The electronic band structure of the Li_5_AuP_2_ compound is obtained along the high-symmetry points in the first Brillouin zone. Figure 9 shows the electronic band structure of the compound at the 20 and 25 GPa, at both of which values this compound is dynamically stable, obtained using the PBE (shown in black) and HSE (shown in red) methods. As it is known, the PBE method tends to underestimate the semiconductors’ electronic band gaps; therefore, the HSE method is employed, instead. The electronic band gaps of the Li_5_AuP_2_ compound are found as 1.05 and 1.30 eV, respectively, using the PBE and HSE methods at 20 GPa, as seen in Figure 9a. The electronic band gaps are also obtained as 1.07 and 1.33 eV, respectively, using the PBE and HSE methods at 25 GPa, as seen in Figure 9b. Other electronic band gaps (0, 5, 10, and 15 GPa) are provided in Figure S4 in the Supporting information. According to that figure, the electronic band gaps determined using the PBE method are 0.79, 0.87, 0.94, and 0.99 eV at 0, 5, 10, and 15 GPa, respectively. There is no need to obtain the electronic band structures for 0, 5, 10, and 15 GPa using the HSE method because of the dynamical instability at these pressure values, not to mention the very lengthy and time-consuming calculations required. For the Li_5_AuP_2_ compound, the pressure increment results in higher band gap values.

The Mulliken population analysis^29^ is performed for the Li_5_AuP_2_ compound using the CASTEP program, which uses the method that was proposed by Sanchez-Portal et al.^30^ In this method, the plane wave basis set transforms to a linear combination of the atomic orbitals to calculate the Mulliken populations and the Mulliken atomic charges. These charges provide an insight into the charge transfer among the atoms in the Li_5_AuP_2_ compound; yet, in general, the numerical results obtained from this method are not consistent with the experimental results in some cases because of the usage of electron wave functions. Therefore, the Hirshfeld charges are also calculated using the Hirshfeld population analysis^31^ that uses well-defined atomic fragments. Table 5 lists the Mulliken atomic populations, Mulliken charges, and Hirshfeld charges for the Li_5_AuP_2_ compound under 20 and 25 GPa values. A positive Mulliken or Hirshfeld charge indicates that the charge is transferred away from the atom, and if it is negative, the charge is received by the atom.^32^ According to Table 5, the Li atoms transfer charge to both Au and P atoms based on the Mulliken population analysis, while the Li and Au atoms transfer charge to the P atoms based on the Hirshfeld population analysis. The results show that the Mulliken charges are higher than the Hirshfeld charges for all the atoms. Table S1 lists the orbital populations, Mulliken and Hirshfeld charges for 0, 5, 10, and 15 GPa pressures. Similar behaviors are observed for these pressures at 20 and 25 GPa.

**Table 5:** Orbital Populations (Electron), Mulliken Atomic Charges (Electron), and Hirshfeld Atomic Charge (Electron) of the Li_5_AuP_2_ Compound at 20 and 25 GPa Values

		Mulliken atomic population					
pressure	species	s	p	d	total	Mulliken charge	Hirshfeld charge
20 GPa	Li1	1.99			1.99	1.01	0.02
	Li2	2.37			2.37	0.63	0.00
	Li3	2.36			2.36	0.64	0.03
	Au	1.13	1.93	9.75	12.81	–1.81	0.29
	P1	1.66	4.40		6.06	–1.06	–0.19
	P2	1.66	4.40		6.06	–1.06	–0.19
25 GPa	Li1	1.97			1.97	1.03	0.02
	Li2	2.37			2.37	0.63	0.00
	Li3	2.36			2.36	0.64	0.02
	Au	1.14	1.98	9.75	12.87	–1.87	0.30
	P1	1.65	4.40		6.05	–1.05	–0.19
	P2	1.65	4.40		6.05	–1.05	–0.19

Table 6 lists the Mulliken bond overlap populations, bond lengths, and total number of bonds for the Li_5_AuP_2_ compound at 0 GPa. Accordingly, there are anti-bondings among the Li–P, Li–Li, and Li–Au atoms. In addition, upon further examining the bond overlap populations, one can see that the P–Au atoms are bonded more covalently than the other atoms in the structure. Table S2 lists the bond overlap populations for other pressures. As can be seen, similar behaviors are observed for these pressures at 20 and 25 GPa.

**Table 6:** Calculated Mulliken Bond Overlap Population of μ-Type Bond *P*^μ^, Bond Length *d*^μ^ (Å), Total Number of μ-Type Bond *N*^μ^, and the Total Number of Bond *N* of the Li_5_AuP_2_ Compound at 20 and 25 GPa Values

pressure	bond	*P*^μ^	*d*^μ^	*N*^μ^	*N*
**20 GPa**	**Li–P**	0.09	2.33	16	192
		0.20	2.42	16	
		–0.22	2.44	16	
		0.62	2.46	8	
		0.28	2.79	8	
		0.17	2.82	16	
	**Li–Li**	–1.59	2.33	16	
		–0.44	2.42	16	
		–0.60	2.44	16	
		–0.17	2.79	8	
		–0.06	2.96	8	
	**Li–Au**	–1.49	2.52	16	
		–0.93	2.66	8	
		–1.38	2.79	8	
	**P–Au**	0.51	2.52	16	
**25 GPa**	**Li–P**	0.08	2.30	16	192
		0.21	2.39	16	
		–0.25	2.42	16	
		0.68	2.42	8	
		0.31	2.76	8	
		0.19	2.79	16	
	**Li–Li**	–1.93	2.30	16	
		–0.56	2.39	16	
		–0.71	2.42	16	
		–0.22	2.76	8	
		–0.08	2.92	8	
	**Li–Au**	–1.72	2.49	16	
		–1.11	2.63	8	
		–1.61	2.76	8	
	**P–Au**	0.50	2.50	16	

**Figure 10:**
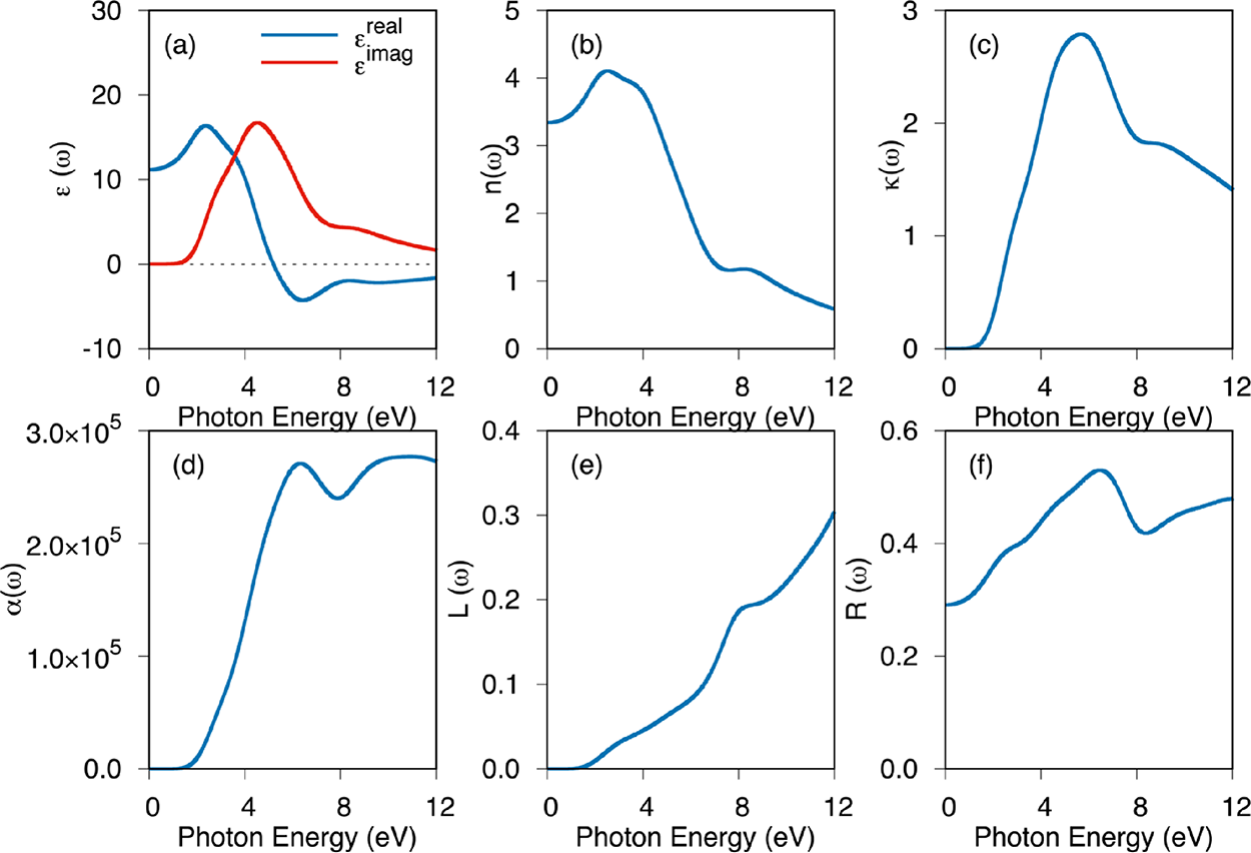
(a) Real and imaginary parts of the dielectric function, (b) refractive index, (c) extinction coefficient, (d) absorption coefficient (in cm^–1^), (e) loss function, and (f) reflectivity of the Li_5_AuP_2_ compound at 20 GPa.

The optical properties of the Li_5_AuP_2_ compound are studied in the 0 to 12 eV range. The complex dielectric function is related to the interactions of photons with ions, which determine how a compound reacts to electromagnetic radiation.^33^ The real and imaginary parts of the dielectric function, as shown in Figure 10 and Figure S5 for 20 and 25 GPa, respectively, are determined using the equation given in ref (34). The real part of the complex dielectric function increases as the energy increases up to about 2.5 eV, as shown in Figure 10 and Figure S5, and it decreases after this energy. The real part of the complex dielectric function intersects with the *x*-axis, and it turns to negative values after about 5.0 eV, indicating a metallic behavior in this energy range. Figure 10 and Figure S2 also show the imaginary part of the complex dielectric function, which takes zero values up to 1.30 and 1.34 eV at 20 and 25 GPa values. This means that the compound is transparent below this energy range. These energy values correspond to the optical band gap of this material and are consistent with the electronic band gaps. Using the formulas given in refs (34, 35), the obtained complex dielectric function is utilized to calculate optical parameters such as the refractive index, extinction coefficient, absorption coefficient, loss functions, and reflectivity, as shown in Figure 10 and Figure S2 at 20 and 25 GPa, respectively. The refractive index takes a 3.34 value at 0 eV energy for both 20 and 25 GPa, and it is known as the “static refractive index”. The behavior of the extinction coefficient is similar to the complex dielectric function, and it is zero up to 1.30 and 1.34 eV for 20 and 25 GPa, respectively. Therefore, they are consistent with the electronic band gaps of this material under these pressure values. The absorption coefficient is related to the amount of light that is absorbed during passing through a material, and, as seen from the figures, the absorption coefficient increases with the rise in the photon energy. The loss function is related to the energy loss of an electron while traversing a material; in case of the Li_5_AuP_2_ compound in this study, this function increases with the rise in the photon energy. The reflectivity is also shown in Figure 10 and Figure S2 at 20 and 25 GPa, respectively. The reflectivity values at zero frequency are 29% for 20 and 25 GPa. It is seen that this value increases by 53% with a rise in the photon energy up to 6.48 eV and 52% up to 6.62 eV for 20 and 25 GPa, respectively. After these energy values, the reflectivity decreases up to 8.39 and 8.61 eV for 20 and 25 GPa, respectively, and it also increases after these energy values up to 12 eV.

## 4 Conclusions

This study investigates the physical properties of the Li_5_AuP_2_ compound under pressure from 0 to 25 GPa using calculations based on DFT. The structural optimizations are performed using the tetragonal crystal structure of this compound. The mechanical and dynamical stabilities reveal that this compound is stable above 18 GPa pressure. The mechanical properties of this compound are also studied, revealing that it has dominantly ionic bonding. The brittle or ductile nature of this compound is shown to change with pressure; it is brittle under 0 and 5 GPa (0, 5, 10, and 15 GPa) pressure and ductile under 10, 15, 20, and 25 GPa (20 and 25 GPa) according to the *B*/*G* ratio (the Cauchy pressure). The anisotropic elastic properties show that the Li_5_AuP_2_ compound is anisotropic with respect to Young’s modulus, linear compressibility, shear modulus, and Poisson’s ratio. The compound is isotropic concerning its linear compressibility under 0 GPa pressure. In addition, the sound wave velocities are visualized in three dimensions. The electronic properties of this compound are obtained along the Brillouin zone using the PBE and HSE functionals, revealing that the compound is a semiconductor. Apart from this, the pressure increment results in higher band gap values for this compound. The Mulliken and bond overlap populations show the chemical nature of the Li_5_AuP_2_ compound where the P–Au atoms are bonded more covalently than the other atoms in the structure. The complex dielectric function of this compound is obtained from 0 to 12 eV. The real part of the complex dielectric function intersects with the *x*-axis, turning to negative after about 5.0 eV and, henceforth, being indicative of metallic behavior in this energy range. On the other hand, the imaginary part of the complex dielectric function is zero up to 1.30 and 1.34 eV at 20 and 25 GPa, respectively, which means that the compound is transparent below this energy range. The optical properties, such as the refractive index, extinction coefficient, absorption coefficient, and loss functions, are determined using the complex dielectric function as well. The refractive index is 3.34 at 0 eV energy for both 20 and 25 GPa. The behavior of the extinction coefficient is similar to the complex dielectric function, and it is zero up to 1.30 and 1.34 eV for 20 and 25 GPa, respectively. Both the absorption coefficient and the loss function increase with the rise of the photon energy.

Recently, it has been predicted in the literature that the Li_5_AuP_2_ compound is stable under 25 GPa pressure. This study investigates this compound using theoretical calculations to reveal in detail its main physical properties under 0 to 25 GPa with 5 GPa intervals. This comprehensive work is expected to guide future studies and to pave the way for different technological applications of this compound.

## Supporting Information Available

The phonon dispersion curves of the Li_5_AuP_2_ compound at 5, 10, 15, and 20 GPa (Figure S1); direction-dependent polycrystalline properties—Young’s modulus, linear compressibility, shear modulus, and Poisson’s ratio—of Li_5_AuP_2_ at 0, 5, 10, and 15 GPa (Figure S2); group velocity, phase velocity, polarization of the sound waves, enhancement factor, and power flow angle of the Li_5_AuP_2_ compound at 25 GPa (Figure S3); electronic band structure of the Li_5_AuP_2_ compound at 0 5, 10, and (d) 15 GPa using the PBE method (Figure S4); orbital populations, atomic Mulliken charges, and Hirshfeld charge of the Li_5_AuP_2_ compound at 5, 10, 15, 20, and 25 GPa (Table S1); calculated Mulliken bond overlap population of the μ-type bond, bond length, total number of μ-type bond, and the total number of bond *N* of the Li_5_AuP_2_ compound at 0, 5, 10, and 15 GPa (Table S2); real and imaginary parts of the dielectric function, refractive index, extinction coefficient, absorption coefficient loss function, and reflectivity of the Li_5_AuP_2_ compound at 25 GPa (Figure S5) (PDF)
