# Supplementary material for: DFT Insights into Noble Gold-Based Compound Li5AuP2: Effect of Pressure on Physical Properties
Source: ACS Omega. 2023 Apr 20;8(17):15673–83. doi: 10.1021/acsomega.3c01217 (PMC10157863; doi:10.1021/acsomega.3c01217)
Supplement: Supplementary file 1 — ao3c01217_si_001.pdf [file ao3c01217_si_001.pdf]

Supporting Information

DFT insights into noble gold-based compound, Li_5AuP_2: effect of pressure on physical properties

Gokhan Surucu[a-*], Aysenur Gencer[b], Ozge Surucu[c], Md. Ashraf Ali[d]

[a]Department of Energy Systems Engineering, Gazi University, Ankara, 06500, Türkiye
[b]Department of Physics, Karamanoglu Mehmetbey University, Karaman 70100, Türkiye
[c]Department of Electrical and Electronics Engineering, Atilim University, Ankara 06836, Türkiye
[d]Department of Physics, Chittagong University of Engineering and Technology (CUET) Chattogram-4349, Bangladesh

*Corresponding Author : Gokhan SURUCU
E-mail address: gokhansurucu@gazi.edu.tr

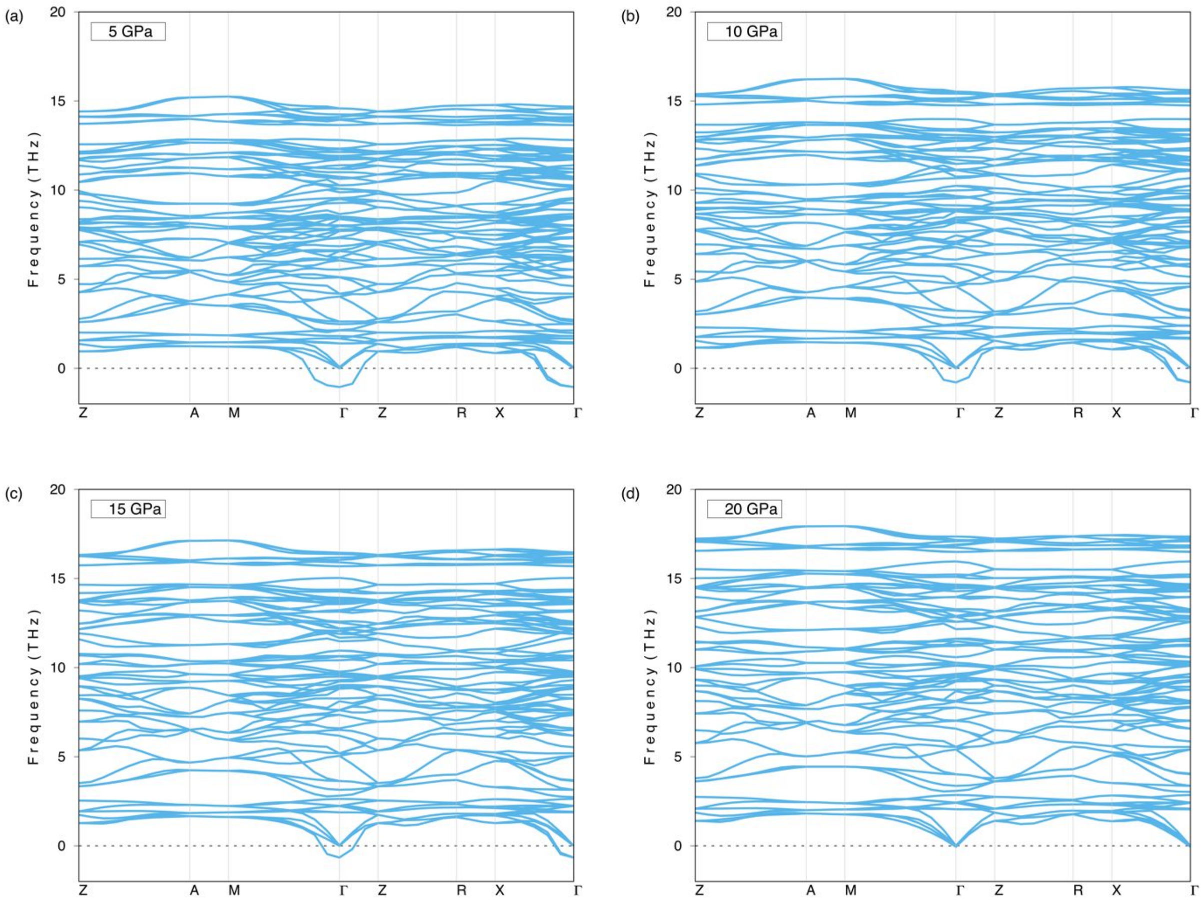

Figure S1: The phonon dispersion curves of Li_5AuP_2 compound at (a) 5 GPa, (b) 10 GPa, (c) 15 GPa, and (d) 20 GPa

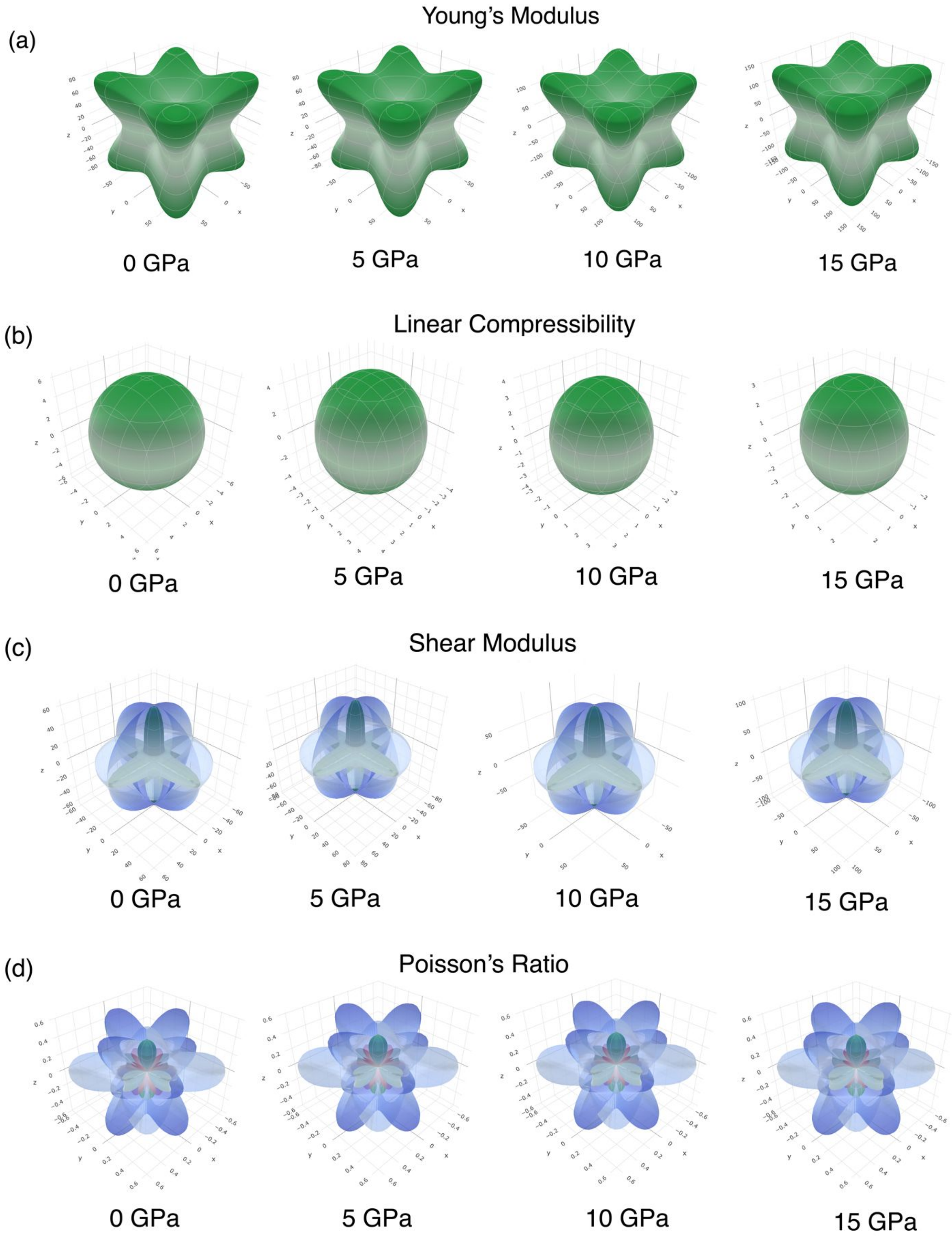
Young's Modulus
(a)
0 GPa
5 GPa
10 GPa
15 GPa
Linear Compressibility
(b)
0 GPa
5 GPa
10 GPa
15 GPa
Shear Modulus
(c)
0 GPa
5 GPa
10 GPa
15 GPa
Poisson's Ratio
(d)
0 GPa
5 GPa
10 GPa
15 GPa

Figure S2: Direction dependent polycrystalline properties - (a) Young's modulus, (b) linear compressibility, (c) shear modulus, and (d) Poisson's ratio - of the Li_5AuP_2 at 0 GPa, 5 GPa, 10 GPa, and 15 GPa

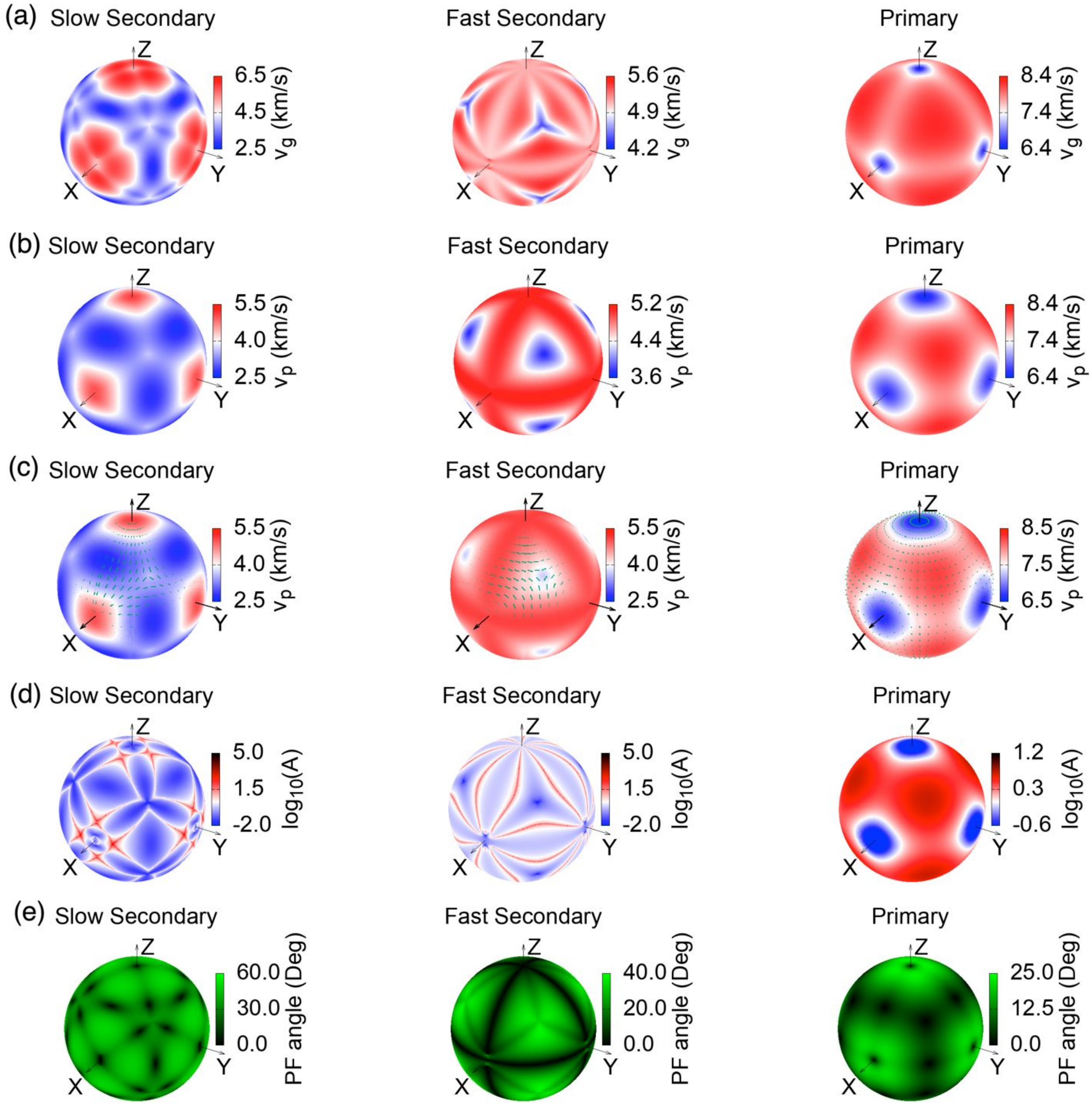

Figure S3: (a) group velocity, (b) phase velocity, (c) polarization of the sound waves, (d) enhancement factor, and (e) power flow angle of the Li_5AuP_2 compound at 25 GPa

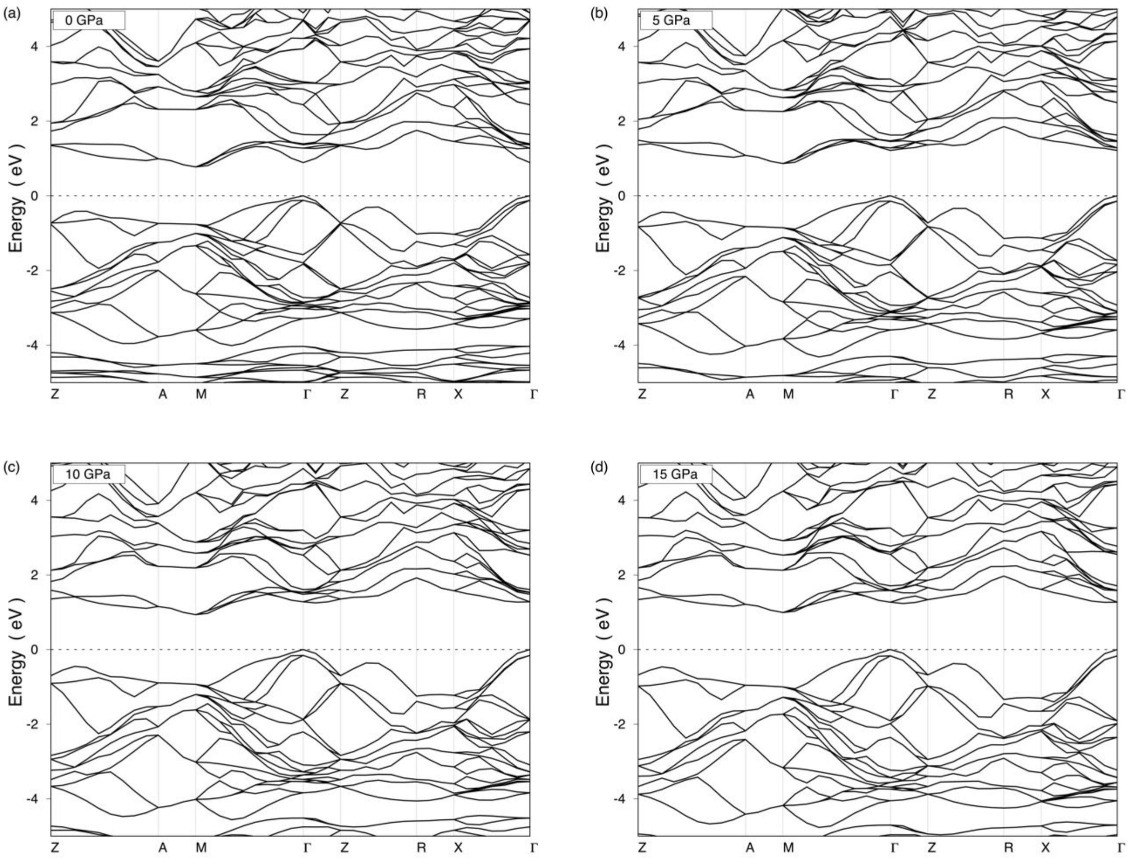

Figure S4: Electronic band structure of the Li_5AuP_2 compound at (a) 0 GPa, (b) 5 GPa, (c) 10 GPa, and (d) 15 GPa using PBE method

Table S1: Orbital populations (electron), atomic Mulliken charges (electron), and Hirshfeld charge (electron) of Li_5AuP_2 compound at 5 GPa, 10 GPa, 15 GPa, 20 GPa, and 25 GPa pressures

**Pressure**	**Species**	**Mulliken atomic population**				**Mulliken Charge**	**Hirshfeld charge**
		s	p	d	total		
0 GPa	Li1	2.11	-	-	2.11	0.89	0.03
	Li2	2.38	-	-	2.38	0.62	0.02
	Li3	2.37	-	-	2.37	0.63	0.04
	Au	1.11	1.62	9.80	12.52	-1.52	0.22
	P1	1.72	4.35	-	6.07	-1.07	-0.18
	P2	1.72	4.35	-	6.07	-1.07	-0.18
5 GPa	Li1	2.08	-	-	2.08	0.92	0.02
	Li2	2.37	-	-	2.37	0.63	0.01
	Li3	2.37	-	-	2.37	0.63	0.03
	Au	1.11	1.72	9.78	12.61	-1.61	0.24
	P1	1.70	4.37	-	6.07	-1.07	-0.18
	P2	1.70	4.37	-	6.07	-1.07	-0.18
10 GPa	Li1	2.04	-	-	2.04	0.96	0.02
	Li2	2.37	-	-	2.37	0.63	0.01
	Li3	2.37	-	-	2.37	0.63	0.03
	Au	1.12	1.80	9.77	12.68	-1.68	0.26
	P1	1.68	4.38	-	6.06	-1.06	-0.19
	P2	1.68	4.38	-	6.06	-1.06	-0.19
15 GPa	Li1	2.02	-	-	2.02	0.98	0.02
	Li2	2.37	-	-	2.37	0.63	0.01
	Li3	2.36	-	-	2.36	0.64	0.03
	Au	1.13	1.87	9.76	12.75	-1.75	0.28
	P1	1.67	4.39	-	6.06	-1.06	-0.19
	P2	1.67	4.39	-	6.06	-1.06	-0.19

Table S2: The calculated Mulliken bond overlap population of μ-type bond P^{μ}, bond length d^{μ} (Å), total number of μ-type bond N^{μ}, and the total number of bond N of Li_5AuP_2 compound at 0 GPa, 5 GPa, 10 GPa, and 15 GPa pressure values

Pressure	Bond	P^{μ}	d^{μ}	N^{μ}	N
0 GPa	Li-P	0.13	2.54	16	168
		-0.09	2.60	16	
		0.16	2.63	16	
		0.36	2.73	8	
		0.19	2.99	8	
	Li-Li	-0.47	2.54	16	
		-0.24	2.60	16	
		-0.07	2.63	16	
		-0.06	2.99	8	
	Li-Au	-0.64	2.68	16	
		-0.33	2.89	8	
		-0.54	2.99	8	
	P-Au	0.50	2.68	16	
5 GPa	Li-P	0.13	2.46	16	184
		-0.12	2.54	16	
		0.17	2.56	16	
		0.43	2.63	8	
		0.21	2.92	8	
		0.11	2.97	16	
	Li-Li	-0.72	2.46	16	
		-0.32	2.54	16	
		-0.15	2.56	16	
		-0.08	2.93	8	
	Li-Au	-0.84	2.63	16	
		-0.47	2.81	8	
		-0.75	2.93	8	
	P-Au	0.50	2.63	16	
10 GPa	Li-P	0.12	2.41	16	184
		-0.16	2.50	16	
		0.18	2.51	16	
		0.50	2.56	8	
		0.24	2.87	8	
		0.13	2.91	16	
	Li-Li	-0.98	2.41	16	
		-0.24	2.50	16	
		-0.40	2.50	16	
		-0.11	2.87	8	

	**Li-Au**	-1.05	2.59	16	
		-0.67	2.75	8	
		-0.95	2.87	8	
	**P-Au**	0.51	2.59	16	
**15 GPa**	**Li-P**	0.11	2.37	16	184
		0.19	2.46	16	
		-0.19	2.47	16	
		0.56	2.50	8	
		0.26	2.83	8	
		0.15	2.87	16	
	**Li-Li**	-1.27	2.37	16	
		-0.33	2.46	16	
		-0.49	2.47	16	
		-0.14	2.83	8	
	**Li-Au**	-1.26	2.55	16	
		-0.77	2.70	8	
		-1.16	2.83	8	
	**P-Au**	0.51	2.55	16	

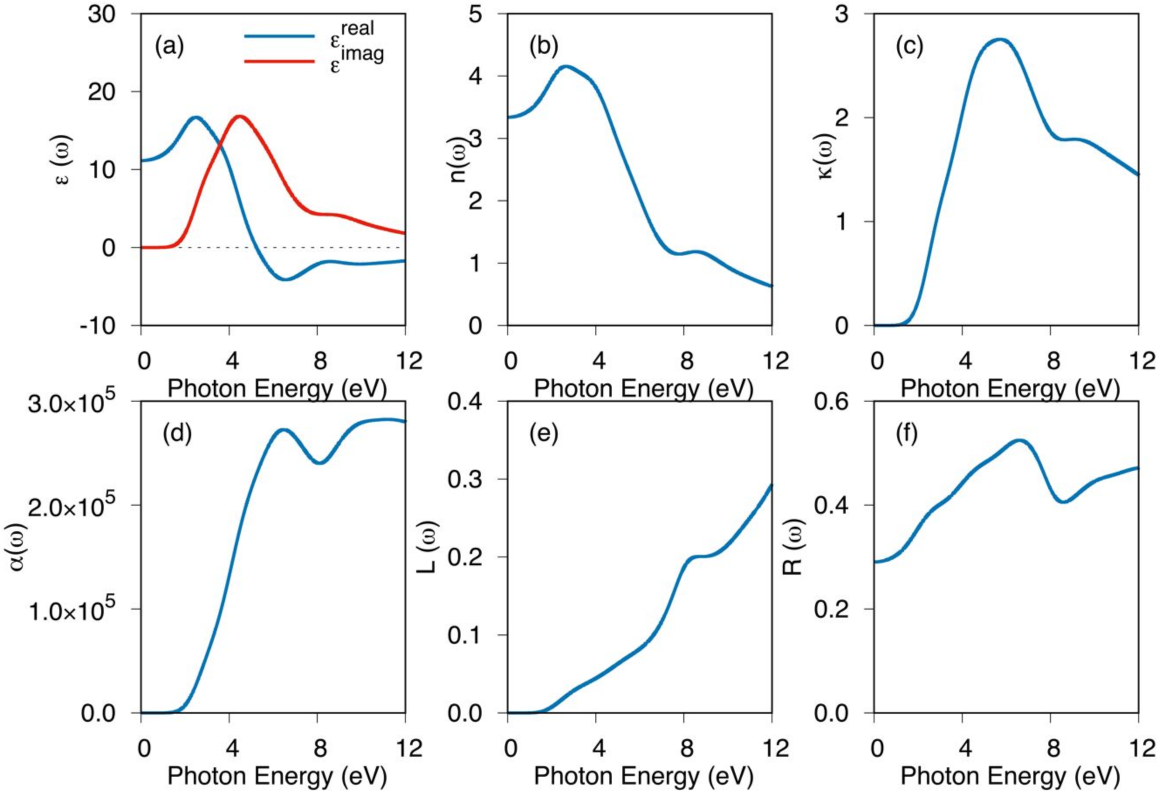

Figure S5: (a) the real and imaginary parts of the dielectric function, b) refractive index, (c) extinction coefficient, (d) absorption coefficient (in cm^{-1}), (e) loss function, and (f) reflectivity of the Li_5AuP_2 compound at 25 GPa
